# A Stretchable and Transparent Electrode for Visual Electrophysiological Acquisition

**DOI:** 10.3390/bios15100701

**Published:** 2025-10-17

**Authors:** Qiwei Dong, Maowen Xie, Mengyao Yuan, Wenhao Lou, Guang Yao, Yuan Lin

**Affiliations:** 1 School of Materials and Energy, University of Electronic Science and Technology of China, Chengdu 611731, China; 2 School of Medicine, University of Electronic Science and Technology of China, Chengdu 610054, China; 3 Medico-Engineering Cooperation on Applied Medicine Research Center, University of Electronic Science and Technology of China, Chengdu 611731, China

**Keywords:** electrophysiological electrode, stretchable and transparent, ophthalmology

## Abstract

Visual impairments pose a significant global health challenge, and visual electrophysiological (EP) acquisition plays a pivotal role in diagnosing ophthalmic diseases. However, traditional electrodes still encounter limitations such as inadequate mechanical adaptability and reusability. This study proposes a stretchable and transparent electrode (STE) consisting of a conductive paste/indium tin oxide layer on a polymethyl methacrylate substrate. Leveraging an island–bridge design, the STE renders reliable performance even after being subjected to 1000 cycles of 25% lateral strain and 18% diagonal strain, exhibiting exceptional mechanical flexibility and realizing seamless attachment to soft tissue. Furthermore, optimized conductive paste layer thickness yields a signal-to-noise ratio comparable to commercial electrodes, achieving equivalent performance to Ag/AgCl electrodes in electroretinogram (ERG), electrooculography (EOG), and visual evoked potential (VEP) acquisition. The STE’s mechanical suitability and inconspicuous features hold significant potential for widespread clinical adoption in ophthalmic diagnostics and personalized eye healthcare, offering improved comfort, reusability, and diagnostic precision.

## 1. Introduction

Vision, responsible for processing over 80% of sensory input, is paramount to human senses [1,2]. The global burden of visual impairment is significant, with projections estimating 834 million affected individuals by 2050, including 61 million experiencing complete blindness [3]. The leading causes of blindness, including retinitis pigmentosa, inflammatory chorioretinopathy, and glaucoma, underscore the urgent need for early diagnosis and timely intervention [4,5,6]. Visual electrophysiological (EP) examinations, such as electroretinogram (ERG) [7], electrooculography (EOG) [8], and visual evoked potential (VEP) [9], have emerged as indispensable tools for identifying visual impairments by capturing precise electrical signals reflecting ocular health [10]. In addition, as the demand for longitudinal monitoring grows and ophthalmic monitoring platforms continue to mature, sustaining stable, long-duration recordings has become essential [10,11,12]. These EP recordings rely critically on electrodes, the interface responsible for signal acquisition, where stable electrode–skin contact and low interface impedance are prerequisites for high-quality and long-term EP acquisition.

EP acquisition electrodes are generally classified as minimally invasive or non-invasive. Minimally invasive electrodes—such as microneedle arrays, modulus-adjustable dry microneedles, and multichannel microneedle patches—achieve low interface impedance by gently penetrating the stratum corneum to establish direct epidermal contact, thereby enabling high-fidelity signals, stable coupling, and reduced motion/hair interference for long-duration, precise recordings [13,14,15]. Unfortunately, tissue penetration introduces safety and comfort concerns: even shallow insertion can cause irritation, erythema, rash, or pain, and repeated or prolonged use may increase the risks of inflammation or infection; in addition, accurate placement and ongoing maintenance complicate routine use, restricting broader deployment despite clear gains in signal quality [16,17]. Non-invasive acquisition electrodes are primarily categorized into wet or dry. Ag/AgCl wet electrodes, widely adopted in clinical practice due to their excellent electrical properties, provide reliable performance for short-term recordings [18]. However, their efficacy diminishes with dehydration, leading to increased impedance and a compromised signal-to-noise ratio (SNR), which undermines data reliability during long-term use [19]. Dry electrodes offer a potential solution by eliminating dehydration [20]. However, traditional metal dry electrodes, exhibiting a high elastic modulus, struggle to conform to the irregular surface of soft tissue [21]. Furthermore, combined with inherently higher electrode–skin impedance, this configuration can lead to impedance mismatch and suboptimal EP recording quality [22].

**Figure 1:**
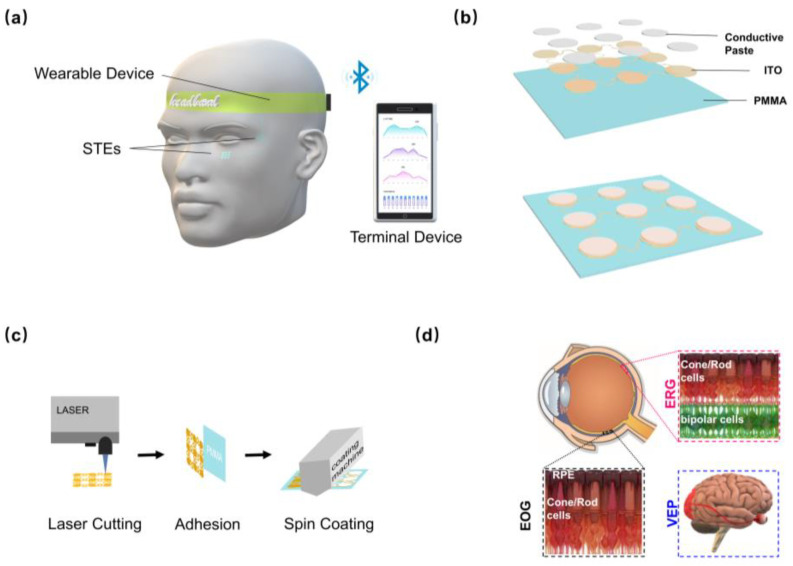
STE preparation and acquisition principle. (**a**) Device overview: STEs are adhered to key skin locations. Physiological electrical signals are then collected and transmitted to a terminal for processing via a wearable device. (**b**) Three-layer structure of the STE: a PMMA substrate, an ITO layer, and a conductive paste layer. (**c**) STE preparation process: The process involves laser-patterning of the ITO film, adhesion to the PMMA substrate, and screen-printing of the conductive paste. (**d**) Physiological principles of ERG, EOG, and VEP acquisition: ERG evaluates retinal electrical activity, EOG measures electrical potentials at the medial and lateral canthi of the eyes, and VEP assesses the visual pathway’s response to visual stimuli. STE: stretchable and transparent electrode, ITO: indium tin oxide, PMMA: polymethyl methacrylate, ERG: electroretinogram, EOG: electrooculography, VEP: visual evoked potential.

This study proposes a novel stretchable and transparent electrode (STE) comprising a conductive paste/indium tin oxide (ITO) layer on a polymethyl methacrylate (PMMA) substrate. This design incorporates an island–bridge structure, which ensures robust performance, maintaining functionality even after 1000 cycles of 25% lateral strain and 18% diagonal strain. The STE’s exceptional mechanical flexibility facilitates seamless contact with soft tissue, making it ideally suited for continuous, long-term monitoring. Moreover, optimizing the conductive paste layer thickness (300 μm) allows the STE to achieve SNR performance comparable to traditional Ag/AgCl electrodes in ERG, EOG, and VEP recordings. STE’s unique flexibility, durability, and high signal quality make it a promising candidate for ophthalmic diagnostic applications. Its transparent design minimizes visual obstruction, potentially improving patient comfort and acceptance. As shown in Figure 1a, the STE can be adhered to the skin at key locations, with physiological electrical signals collected and transmitted for processing via a wearable device. Ultimately, the STE enhances eye care by providing a reliable and non-invasive method for assessing visual function in both clinical and home settings.

## 2. Materials and Methods

### 2.1. Fabrication and Characterization of the STE

As illustrated in Figure 1b, the STE was designed with a bridge–island structure. A circular unit array provides uniform and large-area electrode coverage, while serpentine interconnects provide adequate extensibility, ensuring optimal electrical performance and structural integrity under tensile strain [23]. ITO film, chosen for its excellent conductivity and transparency, facilitates efficient current conduction while maintaining high optical transparency, contributing to the electrode’s overall aesthetic appeal [24]. A flexible PMMA substrate, selected for its optical transparency and biocompatibility, supports the electrode structure while ensuring its aesthetic qualities. Thus, the combination of circular unit arrays, serpentine interconnects, ITO film, and PMMA allows the STE to achieve large-area coverage, high extensibility, structural stability, and a transparency appearance. The STE fabrication process is depicted in Figure 1c. Initially, a laser micro-machining system (DelphiLaser Inducer-6001-N) was used to finely pattern an ITO film (50 μm, 1 Ω/sq) into nine circular units, each with a 2.1 mm radius and spaced 3 mm apart. Serpentine lines (0.79 mm linewidth, 1.36 mm inner diameter, and 2.94 mm outer diameter) were laser-cut to connect the electrode units. Subsequently, the laser-patterned ITO electrodes were adhered to a 200 μm PMMA tape. Finally, conductive paste (Ten 20, WEAVER) was uniformly applied to the electrode surface using a screen-printing method.

A confocal laser scanning microscope (LSM 800, ZEISS) was employed to acquire three-dimensional images of the STE. A tensile testing machine (IBTC300s, CARE) was used to apply both lateral and diagonal tensile strains to the electrodes, enabling evaluation of their mechanical response under varying strain levels. The lateral strain refers to the tensile strain applied along the sides of the square-shaped STE, while diagonal strain refers to the tensile strain applied along the diagonal direction of the electrode. Simultaneously, impedance measurements were carried out using an impedance analyzer (TH26011BS, Tonghui) to monitor changes in electrical performance during stretching. The electrodes were subjected to repeated lateral and diagonal stretching for 1, 10, 100, and 1000 cycles to assess the effect of repetitive stretching on the stability. To further evaluate the long-term durability and reusability, the STEs were subjected to an extended impedance stability test comprising three repeated cycles. Each cycle consisted of four steps: (i) applying conductive gel onto the electrode surface and measuring the initial skin–electrode impedance; (ii) aging the electrode on a 40 °C hot plate for 8 h to simulate prolonged use; (iii) performing a second skin–electrode impedance measurement; and (iv) immediately removing any residual conductive gel with deionized water. The same four-step procedure was repeated for three consecutive cycles, with skin–electrode impedance measured across the frequency range of 1–100 kHz in each cycle.

### 2.2. Conductive Paste Thickness Optimization

To determine the optimal conductive paste thickness for the STE, we evaluated the SNR of the STE with varying paste thicknesses based on EMG signals. We compared the results to commercial Ag/AgCl electrodes (X-1, XUNDA BRAND). Conductive paste thicknesses of 0, 100, 200, 300, 400, 500, 600, and 800 μm were uniformly applied to the STE surface using a screen-printing machine. The STE was positioned according to the surface electromyography for non-invasive assessment of muscles (SENIAM) project guidelines after pre-cleaning the skin with an alcohol swab [25]. EMG signals were recorded during maximum voluntary contractions of the biceps brachii and brachioradialis muscles using a multi-channel EMG sensor at a sampling rate of 2000 Hz. The signals were amplified and filtered from 20 to 200 Hz. The relative SNR of the EMG was calculated using the following formula [26]:

(1) $$\left\{\begin{array}{c} \mathrm{relative}\;\mathrm{SNR}=\frac{{\mathrm{SNR}}_{\mathrm{STE}}}{\;{\mathrm{SNR}}_{\mathrm{Commercial}\;\mathrm{eletrodes}}}\\ \;\mathrm{SNR}={\left(\frac{A_{\mathrm{Signal}}}{A_{\mathrm{Baseline}}}\right)}^{2} \end{array}\right.$$

where *A_Signal_* and *A_Baseline_* represent the amplitude of signals and baselines.

### 2.3. Clinical EP Examinations

To evaluate the performance of STE in clinical measurements, we compared visual EP examination results obtained with the original STE, the STE subjected to 100 stretches at 25% lateral strain, and traditional Ag/AgCl electrodes. The EP assessments included ERG, EOG, and VEP, with the principles and electrode placement methods illustrated in Figure 1d and Figure S1. Healthy adult participants (aged 18–21 years, all right-handed, *n*= 4–6 participants per test) were recruited. All tests were conducted using an EP testing system (Mon2014D, METROVISION). Prior to testing, the participants’ skin was prepared by cleaning with alcohol to ensure optimal electrode–skin contact. The electrode–skin impedance (ESI) between the electrodes and the skin was maintained below 5 kΩ throughout the experiment to ensure accuracy and stability. For the ERG test, the responses of the rod or cone system (including rod or cone cells and bipolar cells) were recorded under various illumination conditions, with electrodes placed at the temporal and lower eyelid [27,28,29]. The main test types included dark-adapted (DA) 0.01 ERG, DA 3.0 ERG, and light-adapted (LA) 3.0 ERG. Following the International Society for Clinical Electrophysiology of Vision (ISCEV) standards, subjects underwent pupil dilation and 30 min of dark adaptation before recording at flash strengths of 0.01 and 3.0 phot cd∙s∙m^−2^ [29]. Subsequently, participants were light-adapted for 20 min, and LA 3.0 ERG was recorded at a flash strength of 3.0 phot cd∙s∙m^−2^. EOG is used to assess the overall function of the retinal pigment epithelium (RPE). It is important to emphasize that this study primarily focuses on the light peak (LP)/dark trough (DT) ratio, which specifically measures the overall function of the RPE/photoreceptor complex, and the EOG mentioned in this article only focuses on the LP/DA ratio [30]. EOG examinations were conducted after pupil dilation and 30 min of dark adaptation. The first 15 min of the test were performed in the dark, followed by 15 min with a light-adapting background of 100 phot cd∙s∙m^−2^. VEP tests assessed the function of the visual pathway, with electrodes placed on the lower eyelid and occipital scalp [31]. These tests include pattern reversal VEP and flash VEP (used for patients with ocular media opacities or children) with a flash strength of 3.0 phot cd∙s∙m^−2^ [32]. EP test results were analyzed using one-way ANOVA in GraphPad Prism (Prism 8). All clinical procedures and experiments in this study were reviewed and approved by the Ethics Committee of the School of Materials and Energy, University of Electronic Science and Technology of China.

## 3. Results and Discussion

### 3.1. Design and Characterization of the STE

**Figure 2:**
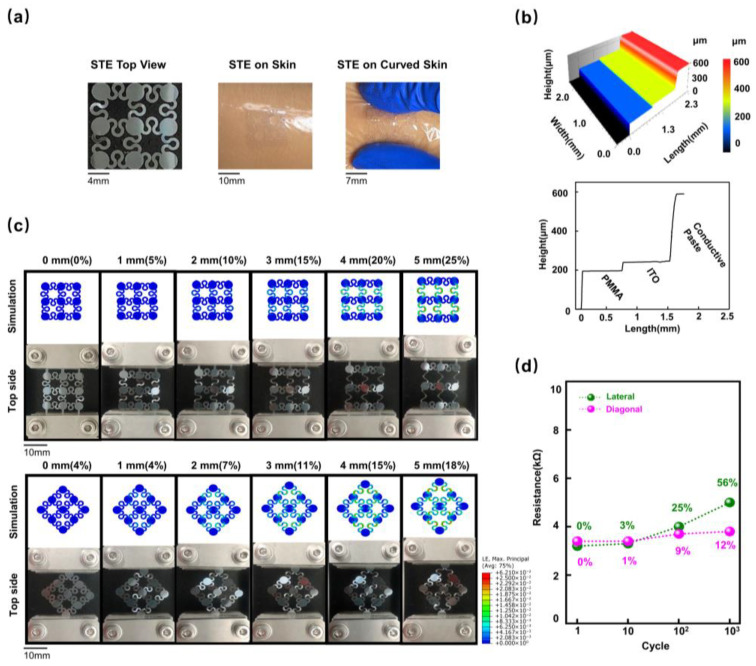
STE characterization. (**a**) Top view of the STE (**left**), skin attachment (**middle**), and flexibility during bending (**right**). This panel demonstrates the overall dimensions, skin attachment, and conformity of the STE during skin deformation. (**b**) Three-dimensional thickness diagrams of the STE (**top**) and corresponding thickness ladder diagram (**bottom**) illustrating the cross-sectional thicknesses of the conductive paste, ITO film, and PMMA substrate. (**c**) Lateral (**top**) and diagonal (**bottom**) tensile testing showing the STE’s mechanical response to lateral and diagonal tensile strains, respectively. (**d**) Resistance stability during repeated stretching. Resistance changes and percentiles of change across 1, 10, 100, and 1000 cycles of 25% lateral strain (green line) and 18% diagonal strain (red line), highlighting the durability and consistency of the STE’s performance. STE: stretchable and transparent electrode; ITO: indium tin oxide; PMMA: polymethyl methacrylate.

The overall dimensions of the STE in the initial state are ~19.5 × 19.5 × 0.55 (L × W × T) mm^3^ (Figure 2a, left). The island–bridge geometry can effectively promote structural robustness, optimize overall modulus, and minimize flexibility restrictions. Figure 2a (middle and right) demonstrates the experimental wearing mode for STE, indicating that the electrode could be seamlessly attached to the skin and subjected to considerable deformation. We employed a three-dimensional microscope to detect the height information of the multi-layered components (Figure 2b). The cross-sectional height profile was intercepted along a scanning line to quantify the multilayer geometry. The overall thickness of the STE is ~550 μm, and the thicknesses of the conductive paste, the ITO film, and the PMMA substrate are 300, 50, and 200 μm, respectively (Figure 2b).

Finite element analysis (FEA) and experimental results of the STE under a series of lateral and diagonal tensile distances are shown in Figure 2c. FEA results of STEs under 0 to 5 mm tensile deformation (lateral strain was 25%, and diagonal strain was 18%) demonstrated that the strain was evenly distributed on the island–bridge blocks. A commercial tensile system was then used to test the corresponding experimental mechanical response of the stretchable STEs (Supplementary Figure S2). Similar deformation behaviors were observed in FEA and experimental results. The strain subjected by the mechanical structure was consistently smaller than the failure strain (3%), showing their good stretchability within the designed deformation range. Long-term electrical performance was assessed using different tensile cycles (Figure 2d). The results showed good device integrity without any observable structural defects and performance degradation after 100-cycle deformation, and the resistance did not exceed 5 kΩ, meeting the ISCEV standards [29]. The minimal resistance fluctuations demonstrated the STE’s outstanding durability and long-term operational suitability for visual EP acquisition.

To further confirm the long-term stability and reusability, an extended impedance durability test was performed (Supplementary Figure S3). The 10 Hz electrode–skin impedance remained below 5 kΩ for up to 24 h and below 7.5 kΩ after 8 h, with no observable degradation after three repeated test cycles. These results validate the STE’s high durability, reusability, and suitability for continuous or long-term electrophysiological monitoring in wearable applications.

### 3.2. Effect of Conductive Paste Thickness on SNR

**Figure 3:**
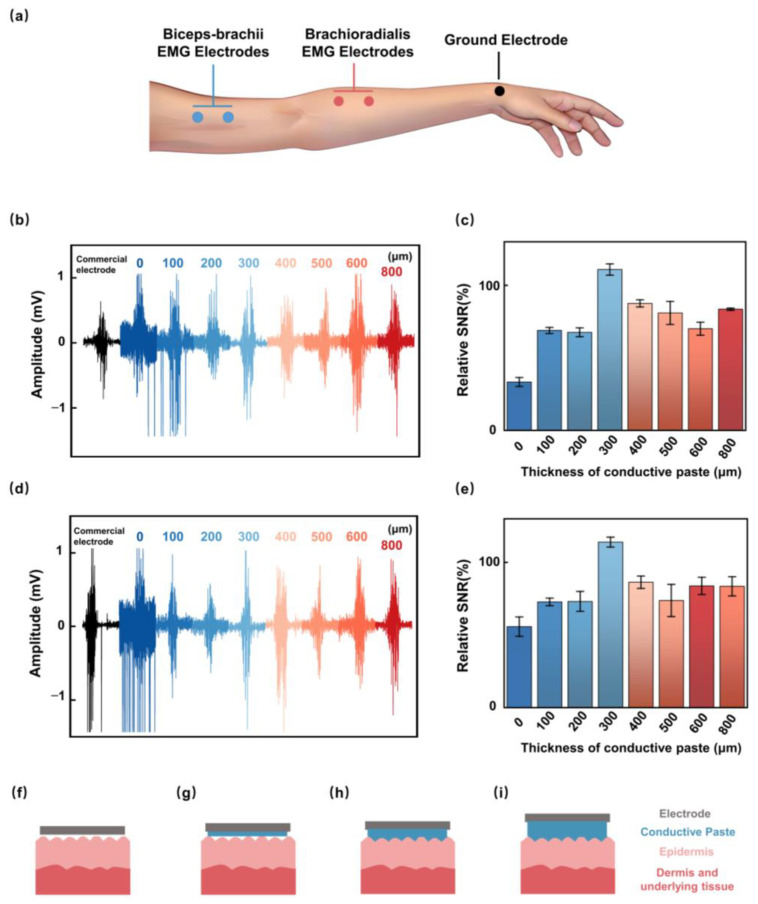
Effects of conductive paste thicknesses on electrical signal acquisition. (**a**) EMG signal acquisition setup. Electrode placement on the biceps brachii and brachioradialis muscles for recording EMG signals under varying conductive paste thicknesses. (**b**) Recorded EMG waveforms from the biceps brachii under different paste thicknesses, illustrating the impact on SNR. (**c**) Comparison of relative SNR (biceps brachii) for varying conductive paste thicknesses. (**d**) Recorded EMG waveforms from the brachioradialis under different paste thicknesses. (**e**) Comparison of relative SNR (brachioradialis) for varying conductive paste thicknesses. (**f**) Schematic of the electrode model without conductive paste, resulting in high impedance and poor SNR. (**g**) Schematic of the electrode model with insufficient conductive paste, leading to suboptimal SNR due to skin–electrode gaps. (**h**) Model with optimal conductive paste: schematic showing the electrode model with the optimal amount of conductive paste, providing low impedance and high SNR. (**i**) Schematic of the electrode model with excessive conductive paste, potentially causing electrode shorting and reduced SNR due to increased impedance. EMG: electromyography; SNR: signal-to-noise ratio.

To verify STE’s EP recording performance, we first systematically evaluated the effect of conductive paste thickness on SNR in the EMG test. First, conductive paste thickness was designed to be 0, 100, 200, 300, 400, 500, 600, and 800 μm to adjust STE’s ESI properties. Subsequently, we investigated the EMG recording performance from the biceps-brachii and brachioradialis during maximum voluntary contraction. Three STEs were employed in the experimental setup for EMG acquisition, where two STEs were placed on the skin of biceps-brachii or brachioradialis, serving as the working and reference electrodes 2 cm apart, and the other one was placed at the wrist as the ground electrode (Figure 3a). EMG signals of biceps-brachii and brachioradialis for different electrodes are depicted in Figure 3b,d, and all curve peaks are located at ~1 mV. When the fist is clenched due to the contraction of the wrist flexor muscles, the muscle electrical signal is immediately observed, and the signal disappears after the hand is relaxed. Relative SNRs extracted from Figure 3b,d are shown in Figure 3c,e, with corresponding values (biceps-brachii SNR and brachioradialis SNR) of paste thickness at 300 μm (110.88%, 113.96%) surpassing (33.34%, 55.69%), (66.86%, 72.71%), (67.58%, 73.03%), (87.53%, 86.24%), (80.96%, 73.69%), (70.08%, 83.74%) and (83.50%, 83.45%) for 0, 100, 200, 400, 500, 600, and 800 μm, respectively.

Supplementary Figure S4 displays the STE sensing mechanism schematic diagram along with its equivalent circuit model. Simultaneously, this study examines the effect of varying conductive paste thickness on SNR, categorizing the results into four distinct models. (i) Without conductive paste (dry contact), large micro-voids exist between the electrode and skin, yielding high contact impedance and low SNR (Figure 3f). (ii) With an insufficient paste film, some voids persist and the stratum corneum is only partially hydrated, so the interface does not reach the well-known low-impedance, stable coupling state and SNR remains suboptimal (Figure 3g) [33,34,35]. (iii) With an adequate, thin, and continuous layer, the added electrolyte fills superficial air gaps and hydrates the stratum corneum, effects that electrical impedance studies show produce a pronounced early drop in skin impedance (void filling); this configuration minimizes contact impedance and yields near-optimal SNR (Figure 3h) [36]. (iv) Beyond this optimum, more paste is not necessarily beneficial: in the skin–electrode equivalent circuit, the paste layer contributes a finite series resistance that increases with layer thickness, which is counterproductive for signal transfer (Figure 3i) [37]. In addition, when electrodes are closely spaced, a large volume of electrolyte-rich paste raises the risk of electrolyte bridging/short-circuit between neighboring electrodes, further degrading data quality [33]. Considering optimal SNR and high-quality EP acquisition, the STE with 300 μm paste thickness was chosen for further visual EP recording.

### 3.3. Clinical EP Measurements

**Figure 4:**
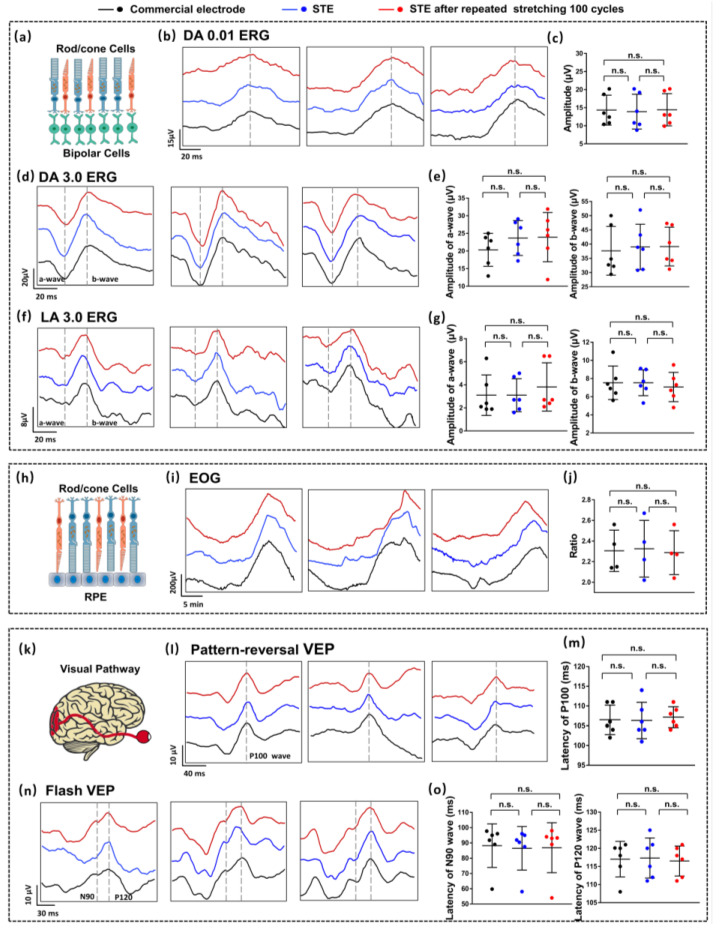
Clinical EP testing. (**a**) Schematic illustrating electrode placement and procedure for ERG, measuring retinal electrical activity in response to light stimuli. (**b**) ERG waveforms recorded under dark-adapted conditions (0.01 cd·s/m^2^) from stretched STE (red) after 100 cycles of 25% lateral strain, unstretched STE (blue), and Ag/AgCl electrodes (black). (**c**) Amplitude measurements from the DA 0.01 ERG tests across electrode types. (**d**) ERG waveforms recorded under dark-adapted conditions (3.0 cd·s/m^2^). (**e**) Amplitude measurements from the DA 3.0 ERG tests. (**f**) ERG waveforms recorded under light-adapted conditions (3.0 cd·s/m^2^). (**g**) Amplitude measurements from the LA 3.0 ERG tests. (**h**) EOG tests to assess the functional connection between RPE and photoreceptor cells. Standard waveforms exhibit a negative dark trough and a positive light peak. (**i**) EOG signals recordings from stretched STE, unstretched STE, and commercial Ag/AgCl electrodes. (**j**) Quantified ratios derived from the EOG tests. (**k**) Schematic illustrating electrode placement and procedure for VEP testing, evaluating the visual pathway’s response to visual stimuli. (**l**) VEP waveforms using a pattern-reversal stimulus, measured from different electrode types. (**m**) Latency measurements extracted from pattern-reversal VEP tests. (**n**) VEP waveforms recorded using a flash stimulus, illustrating the visual pathway’s response to brief visual stimuli. (**o**) Latency measurements extracted from flash VEP tests. Black dots and lines: commercial electrode testing results; blue dots and lines: STE testing results; red dots and lines: STE testing results after 100 stretching cycles. ERG: electroretinogram; EOG: electrooculography; VEP: visual evoked potential; STE: stretchable and transparent electrode; n.s. = no statistical difference, *p* > 0.05.

ERG tests were performed to evaluate the responses of rod, cone, and bipolar cells under varying lighting conditions (Figure 4a) [27]. DA 0.01 ERG represents representative ERG waveforms obtained under dark-adapted conditions with a stimulus intensity of 0.01 cd·s/m^2^, and standard waveforms exhibited identifiable delayed peaks (Supplementary Figure S5a) [30]. Figure 4b and Figure S6a present the amplitudes and latencies of these DA 0.01 ERG delayed peaks obtained from all subjects using the stretched STE (red line), the unstretched STE (blue line), and Ag/AgCl electrodes (black line). One-way ANOVA (Figure 4c and Figure S6b, and Supplementary Table S1) indicated no statistically significant differences in (amplitude, latency) for the three groups, with (14.4 µV, 63.9 ms) for the stretched STE, (13.9 µV, 69.8 ms) for the unstretched STE, and (14.3 µV, 69.3 ms) for the Ag/AgCl electrode, respectively. DA 3.0 and LA 3.0 ERG represent representative ERG waveforms obtained under dark-adapted and light-adapted conditions, with a stimulus intensity of 3.0 cd·s/m^2^. Typical DA 3.0 and LA 3.0 ERG waveforms included an a-wave followed by a b-wave (Supplementary Figure S5b,c). For DA 3.0 ERG, the a-wave and b-wave waveforms are shown in Figure 4d and Figure S6c. Average (amplitude, latency) are (23.9 µV, 24.2 ms), (23.7 µV, 24.5 ms), and (20.3 µV, 25.2 ms) for a-wave waveforms and (39.2 µV, 46.4 ms), (39.0 µV, 47.7 ms), and (37.7 µV, 47.2 ms) for b-wave waveforms, where no significant difference was observed among three groups (Figure 4e and Figure S6d, Supplementary Table S1). Similarly, for LA 3.0 ERG, the a-wave and b-wave are shown in Figure 4f and Figure S6e. The average amplitude and latency for the a-wave are 3.8 µV, 3.1 µV, and 3.1 µV and 20.7 ms, 20.5 ms, and 21.2 ms, respectively and for the b-wave are 7.1 µV, 7.5 µV, and 7.5 µV and 40.2 ms, 40.2 ms, and 40.0 ms, respectively. Statistical analysis revealed no significant differences among the measurements obtained with different electrodes (Figure 4g and Figure S6f, Supplementary Table S1). Even after being subjected to multiple tensile deformations, the STE exhibited consistent key performance metrics compared to commercial electrodes during ERG testing [38].

As shown in Figure 4h, EOG tests were employed to assess the functional connection between the RPE and photoreceptor cells, with standard waveforms featuring a negative dark trough and a positive light peak (Supplementary Figure S5d) [30,32]. The ratio of the light peak to the dark trough served as a key parameter, with average values of 2.29, 2.33, and 2.31 across the stretched STE, unstretched STE, and commercial Ag/AgCl electrode groups, respectively. No statistically significant differences were observed among the groups (Figure 4h–j and Figure S6g, Supplementary Table S1). VEP tests were applied to evaluate the integrity and conduction function of the visual pathway (Figure 4k) [31]. Supplementary Figure S5e illustrates the pattern reversal VEP test, which is characterized by a positive P100 wave around 100 ms, and Supplementary Figure S5f shows that a negative N90 represents the flash VEP wave around 90 ms and a positive P120 wave around 120 ms [32]. The results of the pattern reversal VEP and flash VEP are shown in Figure 4l,n and Figure S6h,j. One-way ANOVA demonstrated no significant differences among the three electrode groups in P100 wave (mean values: 8.7 µV, 107.2 ms, 9.4 µV, 106.4 ms, 8.3 µV, 106.5 ms), N90 wave (mean values: 4.7 µV, 86.9 ms, 4.5 µV, 86.5 ms, 2.7 µV, 88.2 ms), and P120 wave (mean values: 12.4 µV, 116.5 ms, 12.5 µV, 117.4 ms, 10.6 µV, 117.0 ms) (Figure 4m,o and Figure S6k, Supplementary Table S1). These findings confirm that the STE maintains robust signal fidelity, even after extensive mechanical deformation, exhibiting performance metrics comparable to those of commercial electrodes. The minimal differences observed between the unstretched and stretched conditions underscore the long-term stability and reliability of the STE for capturing critical physiological signals. These results highlight the promising potential of the STE for integration into advanced, wearable eye health monitoring and diagnostic systems.

## 4. Conclusions

This study introduces a novel stretchable and transparent electrode comprising a conductive paste and ITO layer on a PMMA substrate, designed with an innovative island–bridge structure. This design confers exceptional mechanical flexibility, enabling seamless contact with soft tissue, and incorporates a transparent ITO layer that minimizes visual obstruction, enhancing aesthetics and practical functionality. After 100 stretching cycles, the STE maintains performance metrics comparable to commercial electrodes, demonstrating high signal fidelity and signal-to-noise ratios equivalent to conventional Ag/AgCl electrodes in ERG, EOG, and VEP recordings. Repeated testing also showed consistently low skin–electrode impedance, confirming the device’s stability and reliability for continuous electrophysiological monitoring. These attributes highlight the STE’s potential as a reliable and non-invasive electrode for continuous ophthalmic diagnostics and advanced wearable eye care applications.

## Data Availability

Dataset available on request from the authors.

## Supplementary Materials

The following supporting information can be downloaded at https://www.mdpi.com/article/10.3390/bios15100701/s1, Figure S1. The positions of the visual EP electrodes. The red box for ERG, blue box for VEP, black box for EOG, and the triangle is the grounding electrode. EP: electrophysiological, ERG: electroretinogram, EOG: electrooculography, VEP: visual evoked potential.; Figure S2: Tensile experiments. (a) Photo of the tensile tester. (b) Top view of the experiments. (c) Side view of the experiments. (d) Simulated (top) and experimental (bottom) images of horizontal stretching at 0 mm, 0.5 mm, 1 mm, 1.5 mm, 2 mm, 2.5 mm, 3 mm, 3.5 mm, 4 mm, 4.5 mm, and 5 mm. (e) Simulated (top) and experimental (bottom) images of diagonal stretching at 0 mm, 0.5 mm, 1 mm, 1.5 mm, 2 mm, 2.5 mm, 3 mm, 3.5 mm, 4 mm, 4.5 mm, and 5 mm; Figure S3. Three-cycle gel/heat test of STE and skin–electrode impedance stability. (a) Photographs of STE across cycles 1–3 (Each cycle comprised: (i) applying conductive gel to the electrode and measuring skin–electrode impedance; (ii) aging on a hot plate at 40 °C for 8 h; (iii) a second impedance measurement; and (iv) immediate removal of all conductive gel using deionized water). Three cycles were performed; three electrodes were tested (N = 3). (b) Skin–electrode impedance (Ω) from 1 Hz to 100 kHz for each cycle. (c) Skin–electrode impedance (Ω) at representative frequencies 1, 10, 100, 1 k, 10 k, and 100 k Hz; Figure S4: Electrode sensing schematic model. (a) Schematic diagram of the electrode sensing principle model. (b) Equivalent circuit mode; Figure S5: The ISCEV standard waveforms. Standard waveforms of DA 0.01 ERG (a), DA 3.0 ERG (b), LA 3.0 ERG (c), EOG (d), pattern-reversal VEP (e), and flash VEP (f).; Figure S6: Clinical vision EP testing. (a) DA 0.01 ERG signals (red line for 100 cycles lateral strain stretching STE, blue line for unstretched, and black line for Ag/AgCl commercial electrodes). (b) Latencies extracted from DA 0.01 ERG tests (the red points for 100 cycles stretching STE, the blue points for unstretched STE, and the black points for Ag/AgCl commercial electrodes). (c) DA 3.0 ERG signals. (d) Latencies extracted from DA 3.0 ERG tests. (e) LA 3.0 ERG signals. (f) Latencies extracted from LA 3.0 ERG tests. (g) EOG signals. (h) Pattern-reversal VEP signals. (i) Amplitudes extracted from pattern-reversal VEP tests. (j) Flash VEP signals. (k) Amplitudes extracted from flash VEP tests. (n.s.= no statistical difference, *p* > 0.05); Figure S7: Mechanical adaptability of STE under elbow flexion. (a) Schematic of elbow flexion at 0°, 30°, 60°, 90°, and 120°. (b) Photographs of the electrode at the corresponding bending angles (0°, 30°, 60°, 90°, 120°); Table S1: *p*-value of visual EP examinations.
