# Supplementary material for: A Stretchable and Transparent Electrode for Visual Electrophysiological Acquisition
Source: Biosensors (Basel). 2025 Oct 17;15(10):701. doi: 10.3390/bios15100701 (PMC12562454; doi:10.3390/bios15100701)
Supplement: Supplementary file 1 [file biosensors-15-00701-s001.zip › biosensors-3884415-supplementary.pdf]

biosensors

MDPI

Supplementary Materials

A Stretchable and Transparent Electrode for Visual Electrophysiological Acquisition

Qiwei Dong [1,2,3], Maowen Xie [1], Mengyao Yuan [1], Wenhao Lou [1], Guang Yao [1,3,*] and Yuan Lin [1,3,*]

[1] School of Materials and Energy, University of Electronic Science and Technology of China, Chengdu 611731, China; qw_dong@ibms.pumc.edu.cn (Q.D.); xiemaowen@cqepc.edu.cn (M.X.); nclear635@163.com (M.Y.); wl1024710@gmail.com (W.L.)
[2] School of Medicine, University of Electronic Science and Technology of China, Chengdu 610054, China
[3] Medico-Engineering Cooperation on Applied Medicine Research Center, University of Electronic Science and Technology of China, Chengdu 611731, China
* Correspondence: gyao@uestc.edu.cn (G.Y.); linyuan@uestc.edu.cn (Y.L.)

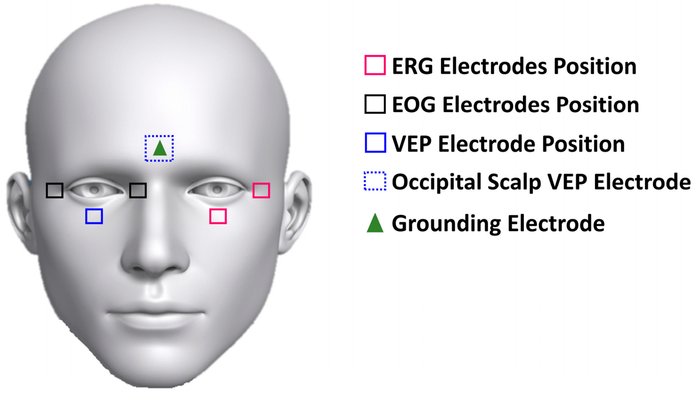

Supplementary Figure S1. The positions of the visual EP electrodes. The red box for ERG, blue box for VEP, black box for EOG, and the triangle is the grounding electrode. EP: electrophysiological, ERG: electroretinogram, EOG: electrooculography, VEP: visual evoked potential.

Received: 4 September 2025
Revised: 7 October 2025
Accepted: 15 October 2025
Published: date

Citation: Dong, Q.; Xie, M.; Yuan, M.; Lou, W.; Yao, G.; Lin, Y. A Stretchable and Transparent Electrode for Visual Electrophysiological Acquisition. *Biosensors* **2025**, *15*, x. https://doi.org/10.3390/xxxxx

Copyright: © 2025 by the authors. Submitted for possible open access publication under the terms and conditions of the Creative Commons Attribution (CC BY) license (https://creativecommons.org/licenses/by/4.0/).

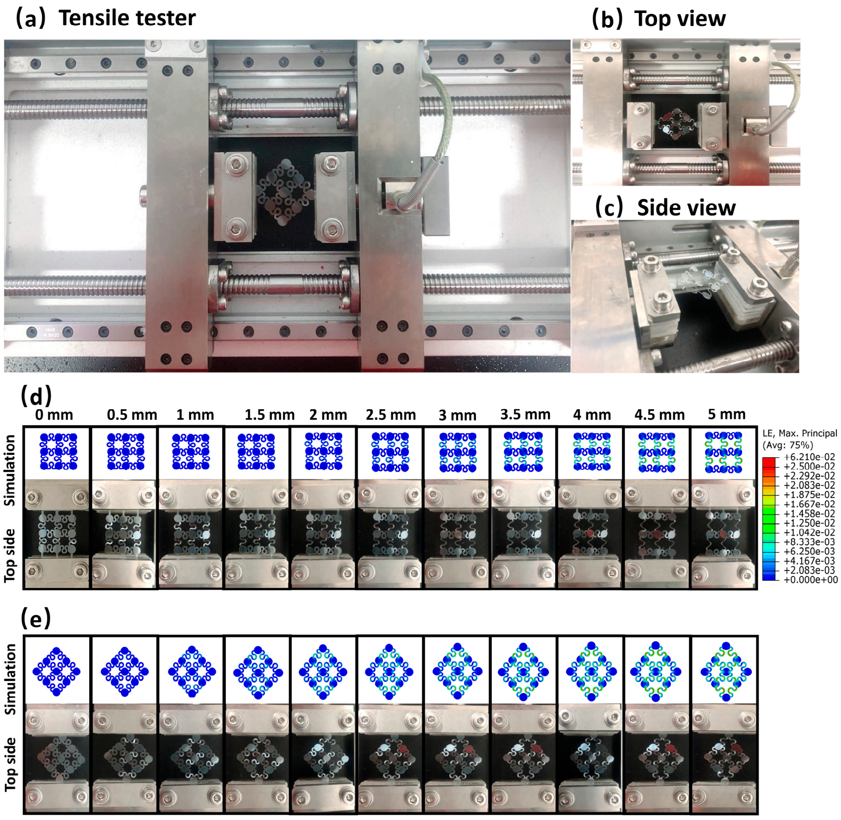

Supplementary Figure S2. Tensile experiments. (a) Photo of the tensile tester. (b) Top view of the experiments. (c) Side view of the experiments. (d) Simulated (top) and experimental (bottom) images of horizontal stretching at 0 mm, 0.5 mm, 1 mm, 1.5 mm, 2 mm, 2.5 mm, 3 mm, 3.5 mm, 4 mm, 4.5 mm, and 5 mm. (e) Simulated (top) and experimental (bottom) images of diagonal stretching at 0 mm, 0.5 mm, 1 mm, 1.5 mm, 2 mm, 2.5 mm, 3 mm, 3.5 mm, 4 mm, 4.5 mm, and 5 mm.

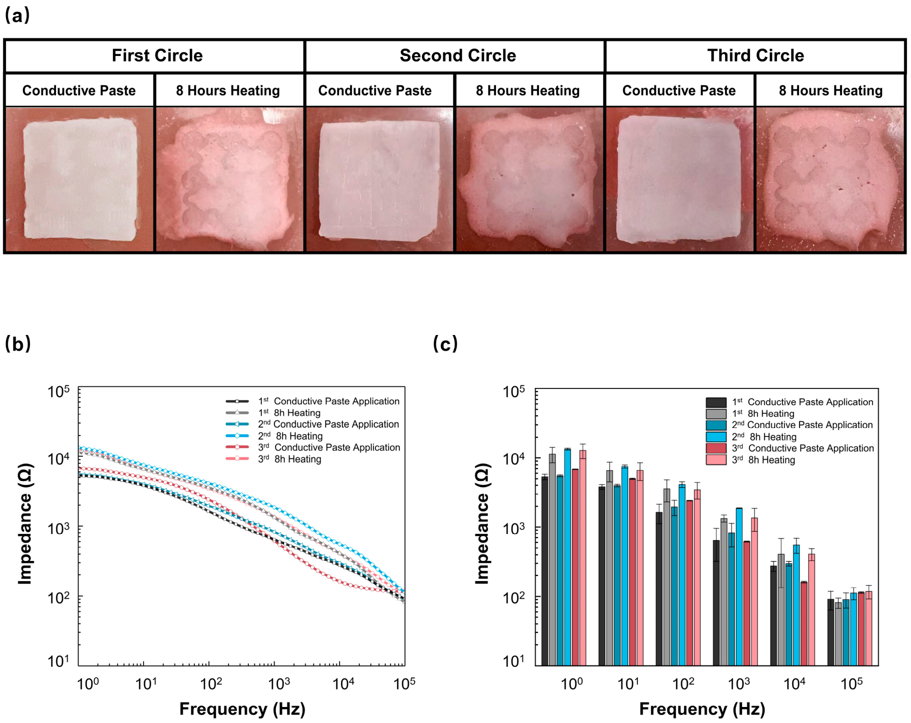

Supplementary Figure S3. Three-cycle gel/heat test of STE and skin–electrode impedance stability. (a) Photographs of STE across cycles 1–3 (Each cycle comprised: (i) applying conductive gel to the electrode and measuring skin–electrode impedance; (ii) aging on a hot plate at 40 °C for 8 h; (iii) a second impedance measurement; and (iv) immediate removal of all conductive gel using deionized water). Three cycles were performed; three electrodes were tested (N = 3). (b) Skin–electrode impedance (Ω) from 1 Hz to 100 kHz for each cycle. (c) Skin–electrode impedance (Ω) at representative frequencies 1, 10, 100, 1 k, 10 k, and 100 k Hz.

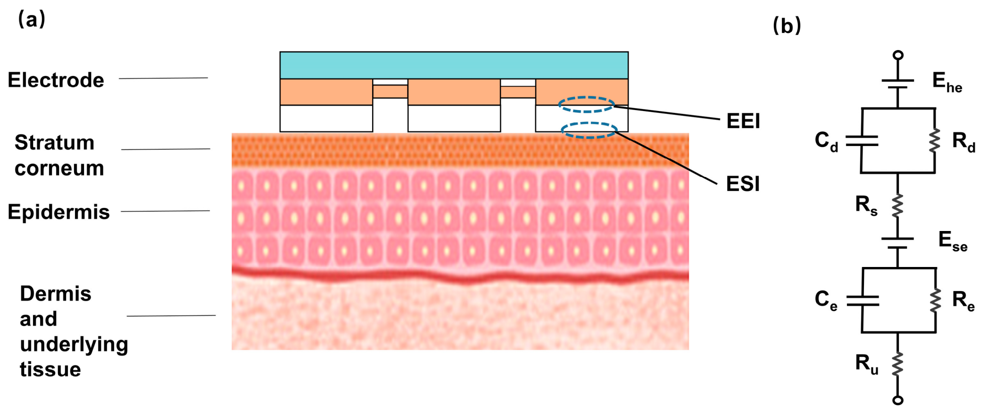

Supplementary Figure S4. Electrode sensing schematic model. (a) Schematic diagram of the electrode sensing principle model. (b) Equivalent circuit model.

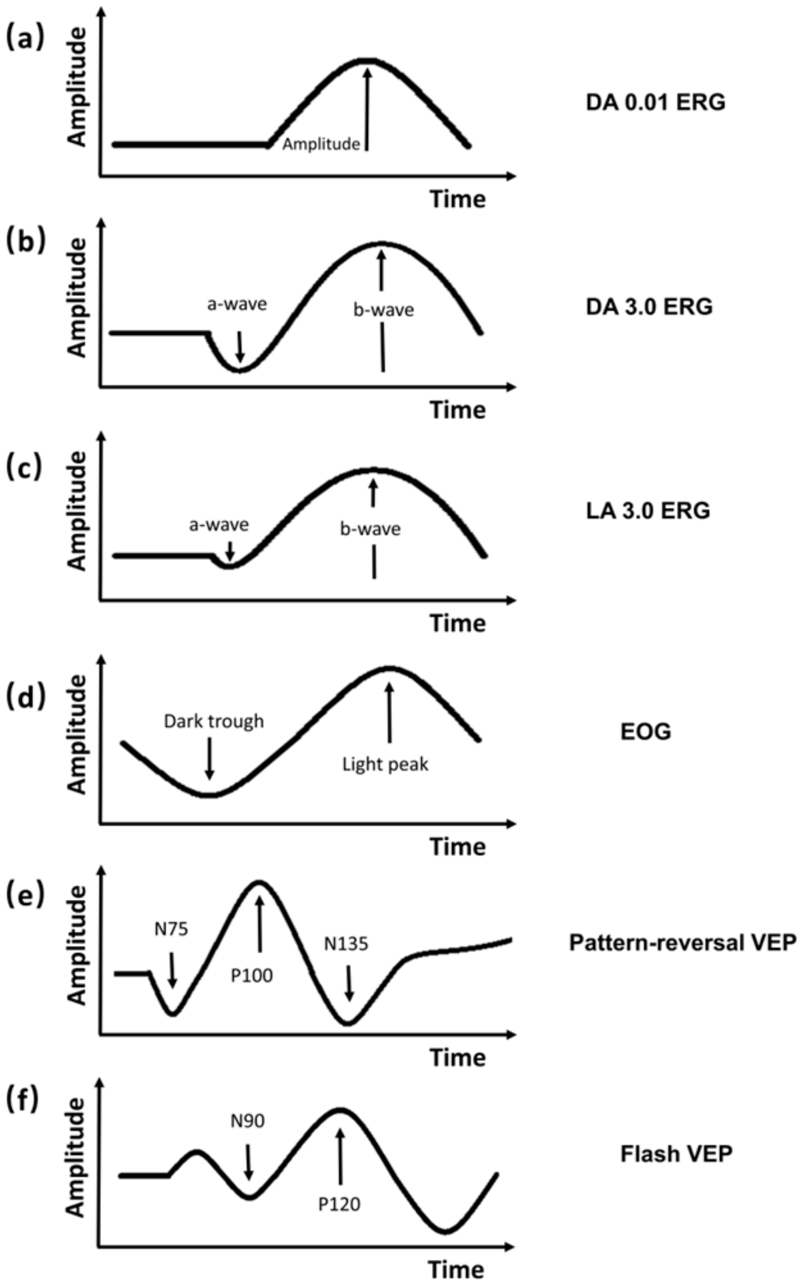

Supplementary Figure S5. The ISCEV standard waveforms. Standard waveforms of DA 0.01 ERG (a), DA 3.0 ERG (b), LA 3.0 ERG (c), EOG (d), pattern-reversal VEP (e), and flash VEP (f).

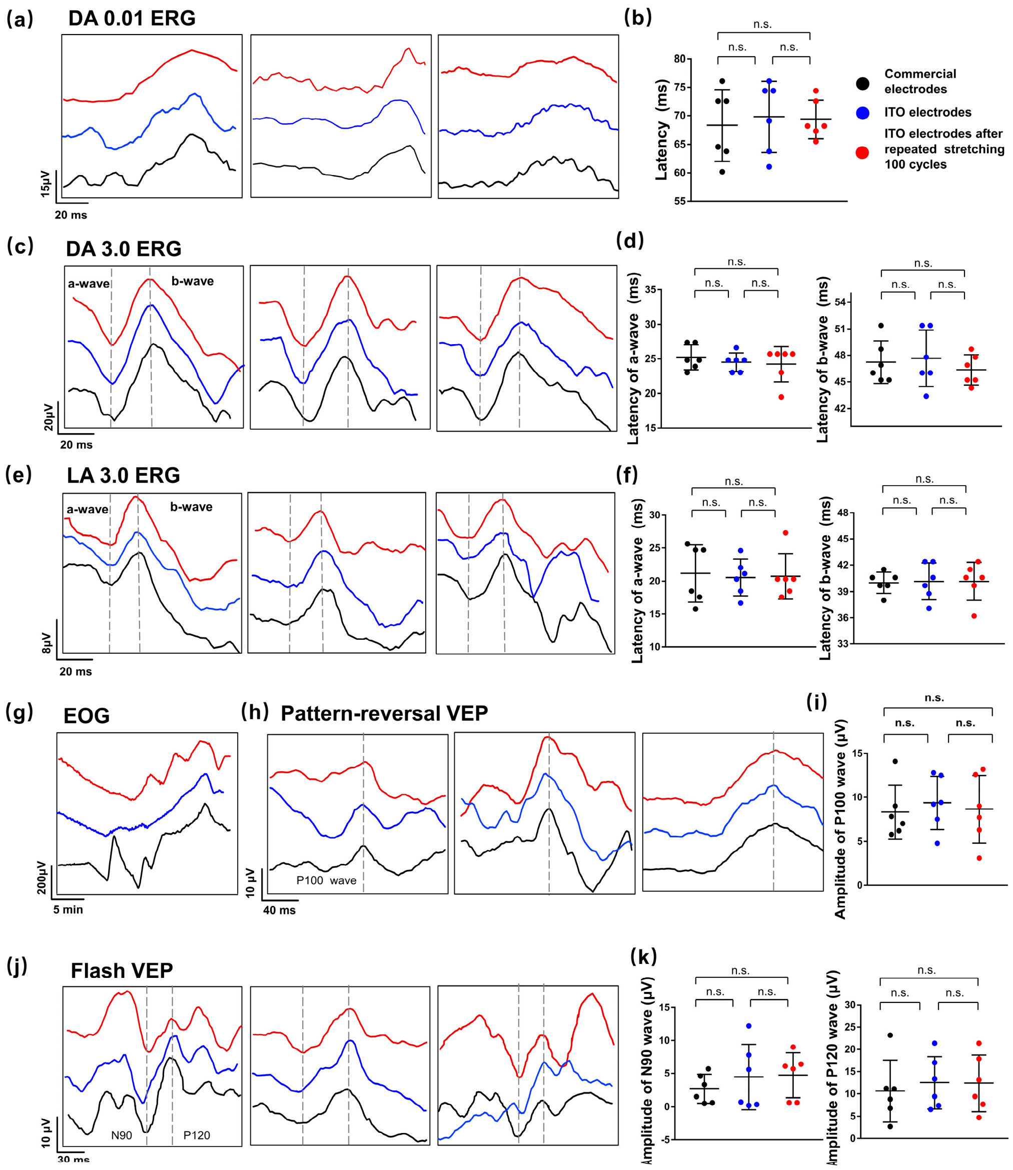

Supplementary Figure S6. Clinical vision EP testing. (a) DA 0.01 ERG signals (red line for 100 cycles lateral strain stretching STE, blue line for unstretched, and black line for Ag/AgCl commercial electrodes). (b) Latencies extracted from DA 0.01 ERG tests (the red points for 100 cycles stretching STE, the blue points for unstretched STE, and the black points for Ag/AgCl commercial electrodes). (c) DA 3.0 ERG signals. (d) Latencies extracted from DA 3.0 ERG tests. (e) LA 3.0 ERG signals. (f) Latencies extracted from LA 3.0 ERG tests. (g) EOG signals. (h) Pattern-reversal VEP signals. (i) Amplitudes extracted from pattern-reversal VEP tests. (j) Flash VEP signals. (k) Amplitudes extracted from flash VEP tests. (n.s.= no statistical difference, $p > 0.05$)

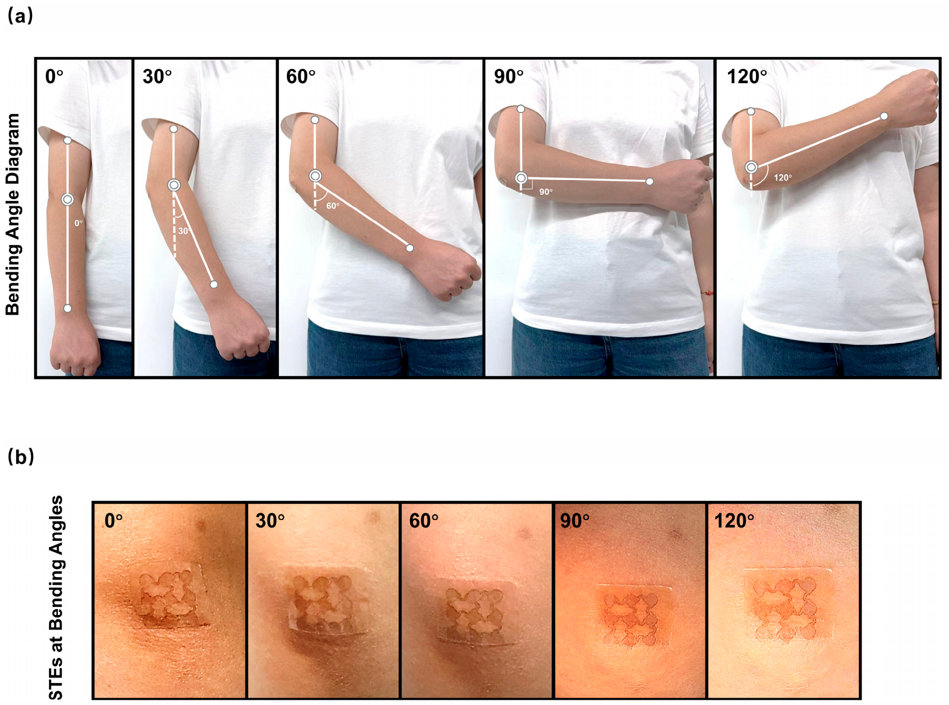

Supplementary Figure S7. Mechanical adaptability of STE under elbow flexion. (a) Schematic of elbow flexion at 0°, 30°, 60°, 90°, and 120°. (b) Photographs of the electrode at the corresponding bending angles (0°, 30°, 60°, 90°, 120°).

Supplementary Table S1. P-value of visual EP examinations

Visual electrophysiological examinations	Compared items	CE vs STE	CE vs SSTE	STE vs SSTE
DA 0.01 ERG	Amplitude	0.8244	0.9600	0.6076
	Latency	0.8212	0.9166	0.9818
DA 3.0 ERG	Amplitude of a-wave	0.1234	0.1561	0.9807
	Latency of a-wave	0.2820	0.5017	0.9168
DA 3.0 ERG	Amplitude of b-wave	0.8632	0.6204	0.9984
	Latency of b-wave	0.9374	0.6566	0.5495
LA 3.0 ERG	Amplitude of a-wave	>0.9999	0.2279	0.2304
	Latency of a-wave	0.9551	0.9528	0.9863
LA 3.0 ERG	Amplitude of b-wave	>0.9999	0.3992	0.7172
	Latency of b-wave	0.9870	0.9697	>0.9999
EOG	Ratio (LA: DT)	0.9815	0.9859	0.9812
Pattern-reversal VEP	Amplitude of P100	0.7140	0.9650	0.5793
	Latency of P100	0.9697	0.7023	0.7340
Flash VEP	Amplitude of N90	0.4743	0.0990	0.9867
	Latency of N90	0.2510	0.4884	0.9365
Flash VEP	Amplitude of P120	0.3483	0.8758	0.9991
	Latency of P120	0.9815	0.8812	0.9295

*CE= Commercial electrodes, STE = Stretchable and Transparent Electrode, SSTE= Stretched STE
